# Hydrogen sulphide modifies the therapeutic potential of bone marrow mesenchymal stem cells in an adjuvant-induced polyarthritis rat model through the mitigation of angiogenesis, ectopic lymphoid tissue formation, and osteoclastogenesis

**DOI:** 10.1007/s10787-025-02078-1

**Published:** 2025-12-18

**Authors:** Sara M. El-Sayed, Mohamed R. Mohamed, Mohamed M. Naguib, Hadeer A. Aglan, Hanaa H. Ahmed

**Affiliations:** 1 Biochemistry Department, Faculty of Science, Ain Shams University, Cairo, Egypt; 2 Hormones Department, Medical Research and Clinical Studies Institute, National Research Centre, Giza, Egypt; 3 Stem Cell Lab., Center of Excellence for Advanced Science, National Research Center, 33 El-Bouhouth St. (Former El-Tahrir St.),Dokki, Giza, 12622 Egypt

**Keywords:** Rheumatoid arthritis, BM-MSCs, NaHS, Conditioned-media, Rats, Naproxen

## Abstract

Among the chronic and progressive autoimmune disorders that primarily affect joints in the hands, wrists, and knees, rheumatoid arthritis (RA) is a highly prevalent one. A significant number of patients develop severe adverse events, display weak responses, or cannot afford long-term use of the current RA medications, requiring more efficient and safer curative alternatives. increasing evidence recommends the application of mesenchymal stem cells (MSCs)-based therapy for mitigating chronic inflammation and boosting tissue renewal in intractable disorders. Moreover, sodium hydrosulphide (NaHS) has recently been found to have anti-inflammatory effects. Therefore, this study compared the therapeutic outcomes of four approaches; bone marrow-derived mesenchymal stem cells (BM-MSCs), their conditioned media (CM), BM-MSCs pre-conditioned with NaHS, and their conditioned media in a rat model of adjuvant-induced polyarthritis. The process involved the isolation of MSCs from rat bone marrow, propagation, and characterization of the isolated cells. polyarthritis was induced in male *Wistar* rats via intradermal injection of type II collagen on day 0 and day 21. Affected rats were treated with naproxen, BM-MSCs, BM-MSCs-CM, NaHS, BM-MSCs preconditioned with NaHS, or BM-MSCs preconditioned with NaHS-CM. The results indicated that the administered cells homed to the bone marrow and bone trabeculae of the knee joint tissue of the afflicted rats. The proposed treatments brought about significant down-regulation of peptidyl arginine deiminase 2 (PAD2) and chemokine ligand 13 (CXCL13) genes as well as angiopoietin-1 (Ang-1) protein expression, along with substantial upregulation of the galectin-1 (GAL-1) gene and osteoprotegerin (OPG) protein expression. Compared with BM-MSCs therapy, the treatment with BM-MSCs preconditioned with NaHS and their CM exhibited superior effect, with values close to those of the controls. In addition, treatment with the CM of BM-MSCs offered a lesser effect compared to BM-MSCs therapy alone. In conclusion, NaHS has the potential to improve the therapeutic capability of BM-MSCs for RA in rats by enhancing their anti-inflammatory, immunomodulatory, and regenerative capacity.

## Introduction

Rheumatoid arthritis (RA) is a chronic systemic autoimmune disease that mainly comprises synovitis and joint injury which are the leading causes of disability (Liu et al. 2024). It is mainfested by synovial inflammatory cells infiltration in the articular cavity and bone corosion caused by the pannus formation (Radu and Bungau 2021). Clinical characteristics of RA are stiffness in the morning, shoulder, neck and pelvic girdle pain in the shoulder, neck, and pelvic girdle pain, fever and loss of mobility, fatigue, malaise, body weight loss, and rheumatoid nodules development. The most important contributors to RA susceptibility are environmental and genetic factors. Other risk factors for RA involve diet, cigarette smoking, hormones, alcohol, microbiota, infection, and coffee (Jahid et al. 2023). Several hypotheses of the pathogenesis of RA have been suggested including the immune abnormalities that can be occured several months to years before the patient becomes symptomatic (Firestein and McInnes 2017). Immune imbalance manifests as dysregulations across various facets of the immune system, spanning both innate and adaptive immunity (Wu et al. 2020). The dysregulated immune system directs its attack towards the synovial membrane, resulting in cartilage degradation, bone corrosion, and joint degeneration (Liu et al. 2019). The presence of inflammation in patients with RA is attributed to cell-mediated immune response including T cells, B cells, and macrophages, alongside the generation of autoantibodies such as rheumatoid factor (RF) and anti-cyclic citrullinated peptide (anti-CCP) antibodies (Kim et al. 2020). The heightened immune activity in RA leads to the anti-inflammatory and proinflammatory cytokines imbalance, sustaining in tissue damage and continual inflammation (Xie et al. 2022).

The global incidence of RA is approximately 1% (Santo et al. 2023) and the global burden of RA has enhanced over the past decades and will continue to increase in the coming years (Cai et al. 2023). The prevalence of RA varies worldwide, with generally higher rates observed in industrialized and urban settings compared to rural and developing regions. This variation may be attributed, at least in part, to diverse risk factors such as genetic predisposition, environmental exposures, hormonal influences, dietary habits, and lifestyle choices (Finckh et al. 2022).

The classification criteria (1987) of the American College of Rheumatology was previously used to enroll RA patients, but due to the shortage of sensitivity in early RA, it was criticized and substituted by the American College of Rheumatology (ACR 2010) criteria, developed by European League Against Rheumatism (EULAR) (Aletaha et al. 2010a). various scoring systems are applied to quantify the activity of RA disease but predominately the disease activity score 28 (DAS 28) is employed. This scoring system depends on 28 counts of the tender and swollen joints (Tamhane et al. 2013). The progression of the disease was identified by radiographs of hands and feet, wherein the bone corrosion degree and narrow space in joints constitutes the loss of cartilage. The association betweeen Doppler ultrasound and MRI edema with erosive radiographic progression of the disease has been reported (Ally 2013). Scintigraphy has a key role in the differential diagnosis of hip and knee joint effusion in RA patients (Sudoł-Szopińska and Ćwikła 2013). Currently, there is no cure available for RA and the treatment approach aims to relieve pain, diminish joint inflammation, inhibit or decelerate joint damage, minimize disability, and sustain patients’ activity levels (Ding et al. 2023).

Nonsteroidal anti-inflammatory drugs (NSAIDs) are commonly prescribed for RA-related pain management, while corticosteroids are utilized for their potent anti-inflammatory properties. Moreover, novel drug targets have been identified to improve RA prognosis, including cell surface molecules, cytokines, and epigenetic regulators (Li and Chen 2022). Disease-modifying antirheumatic drugs (DMARDs) were developed to alter the course, delay progression, inhibit disease activity and retrieve radiographic outcomes (Jahid et al. 2023). Despite these advancements, about 30% of RA patients may exhibit inadequate medication response or intense adverse effects, encompassing bone marrow suppression, hematological abnormalities, liver and/or kidney dysfunction, and susceptibility to infections (Hetland et al. 2020; Kerschbaumer et al. 2020). Given the limitations and potential drawbacks linked with the existing medications, some RA patients may opt against long-term therapy or exhibit poor adherence. Consequently, new therapeutic concepts with enhanced efficacy and tolerability should be developed (Smolen et al. 2020).

Mesenchymal stem cells (MSCs) are a versatile group of adult stem cells that have been extensively studied in preclinical research and are increasingly being explored in clinical trials. MSCs are advantageous for clinical applications due to their low immunogenicity, strong immunomodulatory properties, and the secretion of extracellular vesicles (EVs) containing trophic factors that promote tissue repair and regeneration. Particularly, MSCs can differentiate into various cell types within the mesodermal lineage, making them a promising alternative for treating bone and joint diseases like RA (Shimizu et al. 2022).

Through direct cell-to-cell contact and paracrine signaling, MSCs secrete bioactive molecules that modulate immune responses by suppressing cytotoxic activities of natural killer cells and dendritic cells, thereby inhibiting innate immunity. Moreover, MSCs prevent the proliferation and activities of T helper cells, allow regulatory T cell formation, and secrete factors like indoleamine 2,3-dioxygenase (IDO), prostaglandin E2 (PGE_2_), transforming growth factor-alpha (TGF-α), histocompatibility antigen (HLA)-G5, and interleukin-10 (IL-10) to alleviate inflammation (Ntege et al. 2020). In particular, in the treatment of inflammatory diseases with BM-MSCs, inflammatory signals stimulate these cells to produce growth factors that promote tissue repair, angiogenesis, extracellular matrix remodeling, and tissue progenitor cell differentiation, thereby regulating immune cells within the inflammatory microenvironment (Shi et al. 2018).

Hydrogen sulphide (H_2_S) has emerged as a third gaseous mediator alongside nitric oxide (NO) and carbon monoxide (CO), exerting diverse effects on cellular responses through complex molecular networks. H_2_S functions as an antioxidant and interacts with various signaling molecules (Xu et al. 2019; Fukuto et al. 2020) to modulate processes such as cytoprotection (Zhang et al. 2019), vascular function (Abd Allah et al. 2020), mitochondrial function (Libiad et al. 2019), inflammation (Sun et al. 2020) and tissue repair (Goren et al. 2019a, b). H_2_S donors hold potential as biochemical agents for inducing cartilage and bone repair in arthritis, addressing the destructive effects of chronic inflammation on cartilage integrity (Song et al. 2024). Recent studies have demonstrated the therapeutic efficacy of a combination of H_2_S and BM-MSCs in the treatment of cholestatic liver fibrosis (Mohammed et al. 2021). Also, the pre-conditioning of BM-MSCs with NaHS, as an H_2_S donor, is a promising therapeutic approach against hypoxia-ischemic injury (Zhang et al. 2016). Moreover, the same therapeutic concept showed significant success in rats with heart failure, highlighting the therapeutic potential of H_2_S in conjunction with MSC therapy (Abdelmonem et al. 2019).

The purpose of the present investigation was to explore which one of the applied therapeutic modalities, BM-MSCs, BM-MSCs conditioned media, BM-MSCs pre-conditioned with NaHS, and BM-MSCs pre-conditioned with NaHS-conditioned media could effectively and efficiently alleviate polyarthritis by collagen induced in rats. To fulfill this goal, relevant genes, and protein expression levels were evaluated using real-time quantitative polymerase chain reaction (RT-qPCR) technique and western blot procedure respectively.

## Materials and methods

### In vitro protocol

#### Isolation, propagation, and characterization of BM-MSCs

Bone marrow cells were obtained from 6-week-old male albino *Wistar* rats by flushing the medullary cavity of the excised femur and tibia with Dulbecco’s Modified Eagle’s Medium (DMEM)-high glucose supplemented with 30% fetal bovine serum (FBS). Mononuclear cells were isolated by density gradient centrifugation at ×400 g for 30 min. After three washes with phosphate-buffered saline (PBS), purified cells were cultured in 25 cm^2^ cell culture flasks in a complete culture medium (DMEM-high glucose supplemented with 30% FBS and 1% penicillin/streptomycin) at 37 °C in a humidified incubator with 5% CO_2_. Adherent cells (BM-MSCs) were grown to 80–90% confluence and defined as passage zero (P0) cells. Passage zero cells were washed with PBS, harvested by incubation with 0.25% trypsin-EDTA solution for 5 min at 37 °C, centrifuged at 200 ×g for 10 min, resuspended in complete culture medium, counted, and plated as passage one (P1) cells in cell culture flasks at a density of 1 × 10^6^ cells/flask. The culture medium was refreshed every three days over a 10–14-day period. Cells were passaged by trypsinization till they reached 80–90% confluence (Aglan et al. 2024).

For the characterization of BM-MSCs, cultured cells were morphologically examined using an inverted optical microscope (Olympus, Japan) to verify their identity. In the third passage, cells were immunophenotyped to determine the expression of various cell surface antigens. In brief, harvested cells (0.5 × 10^6^ cells/mL) were stained with a set of monoclonal antibodies, including fluorescein isothiocyanate-conjugated CD45^−^ and CD105^+^ as well as phycoerythrin-conjugated CD90^+^ (Invitrogen, Thermo Fisher Scientific, USA) for 30 min at room temperature in the dark. After the staining step, the cells were washed twice with PBS, resuspended in PBS, and analyzed using a COULTER EPICS XL flow cytometer equipped with System II software (Beckman Coulter, USA), according to the manufacturer’s recommendations (Mahmoud et al. 2020).

#### Preconditioning of BM-MSCs with NaHS

The third passage BM-MSCs were co-cultured with 200 µmol/L NaHS (Sigma-Aldrich, USA) for 30 min before transplantation into rats with RA, as described previously by Xie et al. (2012).

#### Generation of conditioned media

BM-MSCs at the third passage, as well as those preconditioned with NaHS and achieving 80–90% confluence, were rinsed twice with PBS and then cultured in supplemented FBS-free DMEM for 24 h. Subsequently, the medium from an equivalent number of cells from each culture (3 × 10^6^ cells) was gathered and subjected to centrifugation at ×400 g for 20 min (Ionescu et al. 2012).

#### Labeling of BM-MSCs and BM-MSCs preconditioned with NaHS

BM-MSCs at the third passage and those preconditioned with NaHS underwent incubation with ferumoxides injectable solution (Feridex IV, 25 µg/mL, Berlex Laboratories, USA) and poly-L-lysine (PLL, 375 ng/mL, Sigma Aldrich) for 24 h. Feridex was mixed with PLL in a 1 : 10 ratio and shaken for 30 min at room temperature before incubation with cells and then added to the supplemented medium (DMEM high glucose with FBS, 100 U/mL penicillin, and 100 µg/mL streptomycin). Prussian blue staining and eosin counterstaining techniques were utilized to track the iron particles in ferumoxides-labeled BM-MSCs present within knee joint sections, to verify their homing into the knee joints (Balakumaran et al. 2010; Abdel Halim et al. 2021).

### In vivo design

#### Procurement and housing of animals

Sixty-four adult male albino *Wistar* rats, weighing between 140 and 160 g (4 months of age), were provided from the Animal Care Unit at the National Research Centre (NRC) in Giza, Egypt. They were accommodated in polypropylene cages within a well-ventilated room under controlled conditions: an ambient temperature maintained at 25 ± 1 °C, a relative humidity of 55 ± 5%, and a standard 12-hour light-dark cycle. Rats had continuous access to tap water and were fed a standard rodent diet comprising 10% casein, 4% salt mixture, 1% vitamin mixture, 10% corn oil, 5% cellulose, and supplemented to 100 g with corn starch (Meladco Co., Cairo, Egypt). Before experimentation, the rats underwent a one-week acclimatization period in the animal care facility of the Hormones Department, Medical Research and Clinical Studies Institute at the NRC, Giza, Egypt.

#### Ethical approval

All experimental procedures received ethical approval from the Medical Research Ethics Committee of the NRC, Giza, Egypt (Approval ID: 20 062), and adhered strictly to the guidelines outlined by the National Institutes of Health (NIH) concerning the care and usage of laboratory animals, 8th edition, 2011.

#### Induction of polyarthritis model

Rheumatoid arthritis was generated in rats by intradermal administration of 1.5 mg of bovine type II collagen (COL-II, Beijing SEMNL Biotechnology Co., Ltd, China) dissolved in 0.1 M acetic acid and emulsified in complete Freund’s adjuvant (CFA, 1:1, v/v; Sigma Aldrich). Following a three-week interval, rats were given intradermal booster injections of 750 µg of COL-II in incomplete Freund’s adjuvant (ICFA, 1:1, v/v; Sigma Aldrich) (Park et al. 2016).

#### Experimental groups

A total number of 64 rats were randomly assigned into 8 groups, each consisting of 8 rats. The initial group served as the control and was named as Group 1, receiving intradermal injections of acetic acid in two doses; 1.5 mL (first dose) and 0.75 mL (second dose) three weeks apart. Following this, Groups 2 through 8 underwent induction of polyarthritis. After one month, Group 2 remained untreated to serve as the diseased control (polyarthritis untreated). Group 3 received oral naproxen drug (10 mg/kg b.wt.) in 1 mL saline daily for 2 months (Comi et al. 2017) (Naproxen), while Group 4 received a single intravenous infusion of BM-MSCs (5 × 10^6^ cells/rat) in 0.5 mL PBS (Haikal et al. 2019) (BM-MSCs) and Group 5 received intravenous infusion of 0.5 mL BM-MSCs conditioned media (BM-MSCs-CM). Group 6 received intraperitoneal injections of NaHS (1.4 µmol/kg) in 1 mL of saline daily for 2 months (Fang et al. 2009) (NaHS). Group 7 received an intravenous infusion of BM-MSCs preconditioned with NaHS (5 × 10^6^ cells/rat) in 0.5 mL PBS (BM-MSCs-NaHS), and Group 8 received an intravenous infusion of 0.5 mL BM-MSCs preconditioned with NaHS-conditioned media (BM-MSCs-NaHS-CM).

#### Dissection and tissue Preparation

After finalizing the experiment, the rats were anesthetized and sacrificed by cervical dislocation. Both knee joints were excised; the right knee was snap-frozen in liquid nitrogen and stored at − 80 °C for subsequent gene and protein expression analyses, while the left knee was fixed in 10% neutral buffered formalin for 24 h, dehydrated through a graded alcohol, and embedded in paraffin wax for histological investigation.

#### RT-qPCR analysis of PAD2, CXCL13, and GAL-1 gene expression levels in synovial membrane

To evaluate the gene expression levels of peptidyl arginine deiminase 2 (PAD2), chemokine ligand 13 (CXCL13), and Galactin-1 (GAL-1) in synovial membrane knee tissues, quantitative real-time polymerase chain reaction (RT-qPCR) technique was performed. Initially, total RNA was extracted from the synovial membrane using the RNeasy purification reagent (Qiagen, USA) following the manufacturer’s manual. The integrity of the RNA was assessed using the NanoDrop 2000 (Thermo Fisher Scientific, USA) by measuring the 260/280 nm ratio. Subsequently, complementary DNA (cDNA) was synthesized using the high-capacity cDNA synthesis kit (Thermo Fisher Scientific) according to the operating instructions. The quantitative measurement of PAD2, CXCL13, and GAL-1 genes expression was carried out using the QuantiNova SYBR Green PCR kit (Thermo Fisher Scientific) as per the manufacturer’s recommendations. The QuantStudio™ 3 Real-Time PCR System (Thermo Fisher Scientific) was utilized for quantitative real-time analysis. The reaction mixture (25 µl volume) comprised 12.5 µl of QuantiNova SYBR Green PCR kit, 0.75 µl each of forward and reverse primers for the PAD2, CXCL13, and GAL-1 genes (Eurofins, Germany), 100 ng of cDNA template, and RNase-free water. The relative gene expression was determined using the comparative Ct method (2^−ΔΔCt^) (Livak and Schmittgen 2001), with β-actin serving as the endogenous control. The PCR cycling conditions were as follows: an initial denaturation step at 94 °C for 15 min, followed by 40 cycles of denaturation at 94 °C for 15 s, annealing at 55 °C for 30 s, and extension at 72 °C for 30 s. Primer sequences for each gene are presented in Table 1.

**Table 1:** Primer sequences of genes for RT-qPCR

Gene	Primer sequence (5′→ 3′)	References
β –actin	Forward: CCCATCTATGAGGGTTACGC Reverse: TTTAATGTCACGCACGATTTC	Wojcik-Grzybek et al. (2024)
PAD2	Forward: ATTCAAGATAGACCAGGAGGACCAG Reverse: CAGAATAGGAAGGCCAGTGTCAGAA	Ishigami et al. (2001)
CXCL13	Forward: CTGCTCGGAATCTTAGTGT Reverse: GGTAATGCGTCTGCTTCT	Wang et al. (2016)
GAL-1	Forward: ATGGCCTGTGGTCTGGT Reverse: TCACTCAAAGGCCACACACTT	Fan et al. (2018)

#### Western blot analysis of Ang-1 and OPG protein expression levels in synovial membrane

Angiopoietin-1 (Ang-1) and osteoprotegerin (OPG) protein expression levels in the synovial membrane of knee tissues were investigated utilizing the western blot procedure. Initially, protein extraction from tissue was performed using ice-cold RIPA lysis buffer. Quantitative protein analysis was carried out using the Bradford protein assay kit (SK3041) from Bio Basic Inc. (Canada), following the manufacturer’s guidelines. The Bradford assay determined the protein concentration in each sample. Subsequently, equal amounts (20 µg) of proteins were loaded with the equivalent volume of 2x Laemmli sample buffer and boiled at 95 °C for 5 min to ensure protein denaturation before loading onto polyacrylamide gel electrophoresis. Samples were loaded onto TGX Stain-Free™ FastCast™ acrylamide gel (SDS-PAGE) obtained from Bio-Rad Laboratories (USA) and prepared according to the manufacturer’s instructions.

The proteins on the gel were then transferred onto polyvinylidene fluoride (PVDF) membranes using the BioRad Trans-Blot Turbo instrument after blocking with 3% bovine serum albumin (BSA) at room temperature for 1 h in Tris-buffered saline containing 0.1% Tween-20 (TBST; washed 3 times). Following blocking, the membranes were incubated with Rabbit polyclonal Angiopoietin-1 antibody (1:1000; Thermo Fisher Scientific), Rabbit polyclonal Osteoprotegrein antibody (1:2000; Thermo Fisher Scientific), and mouse anti-rat β-actin (1:2000; Thermo Fisher Scientific). After washing with TBST 3 to 5 times, the blots were incubated with horseradish peroxidase-conjugated secondary antibodies rabbit anti-rat IgG (1:2000) (Thermo Fisher Scientific) at room temperature for 1 h. Subsequently, the blots were washed again with TBST 3 to 5 times.

Visualization of the membranes was performed using a standard chemical luminescence method. The chemiluminescent substrate (Clarity™ Western ECL substrate - BIO-RAD, USA) was applied to the blot according to the manufacturer’s recommendation. Equal volumes were added from solution A (Clarity western luminal/enhancer solution) and solution B (peroxidase solution). Chemiluminescent signals were captured using a CCD camera-based imager, and image analysis software was employed to measure the band intensity of the target protein normalized by β-actin on the Chemi Doc MP imager.

#### Histopathology with special stain

After fixation of the knee joints of rats in 10% neutral buffer formalin, then trimmed, tap water was used for washing. The dehydration was done using ascending grades of ethyl alcohol, clearance was performed by xylene and then the samples were embedded in paraffin. The thin section (4–6µ) was processed and stained with Safranin O stain (Bancroft and Gamble 2008) for proteoglycan staining in the cartilage matrix (Schmitz et al. 2010).

### Statistical processing

The experimental results were represented as arithmetic means with their standard errors (S.E). Data were statistically analyzed by one-way analysis of variance (ANOVA) test using the Statistical Package for the Social Sciences (SPSS) 23 followed by determining the least significant difference (LSD) to evaluate the significance between groups. The statistically significant differences were identified at *P* > 0.05.

## Results

### BM-MSCs morphology

**Fig. 1:**
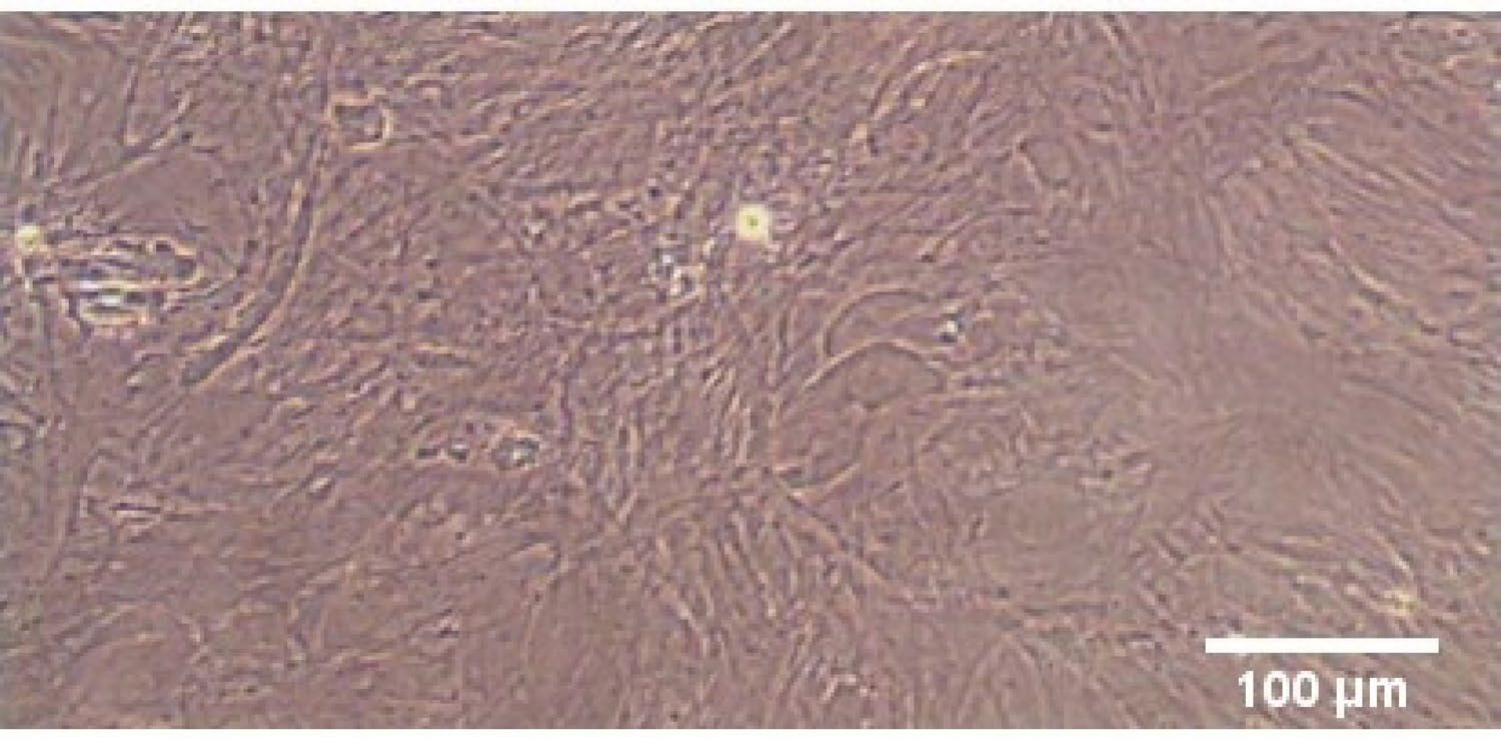
Morphological feature of 3rd passage cultured rat BM-MSCs

The morphology of the 3rd passage BM-MSC population is demonstrated in the photomicrograph presented in Fig. 1. The grown BM-MSCs have clear manifestation of spindle-shaped cells with fibroblastic-like morphology.

### BM-MSCs phenotyping

**Fig. 2:**
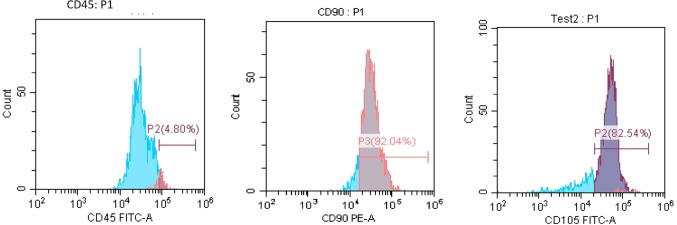
Flow cytometric analysis of rat BM-MSCs surface profile

Flow cytometric analysis for BM-MSCs surface profile was done to confirm whether BM-MSCs maintain their phenotype after culture and expansion. The results revealed that the grown BM-MSCs are positive for CD 90 (92.04%) and CD 105 (82.54%) while they are negative for CD 45 (4.80%) (Fig. 2).

### Homing of infused BM-MSCs and BM-MSCs preconditioned with NaHS

**Fig. 3:**
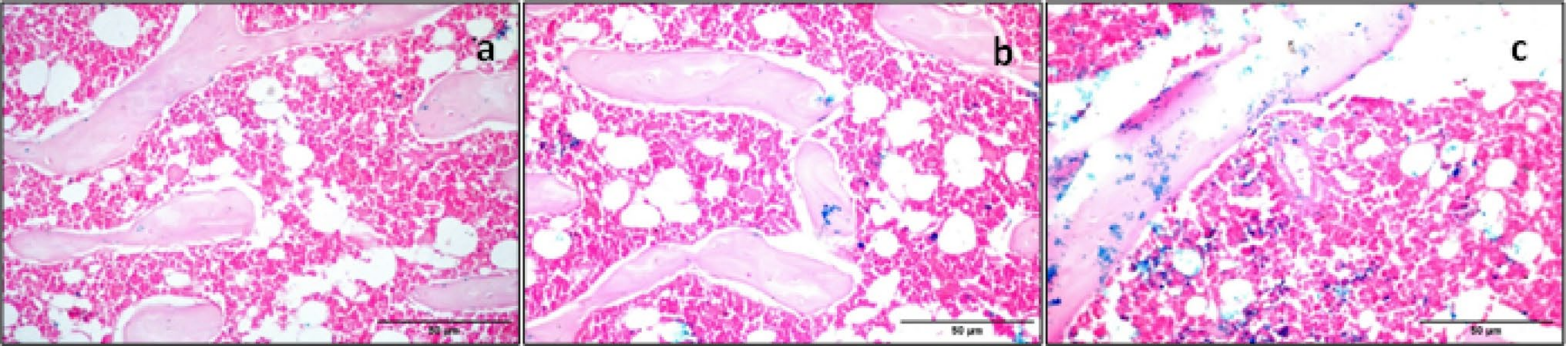
Cross section of Prussian blue stained knee joint tissues. **a** Polyarthritis group treated with BM-MSCs not labeled with ferumoxides, **b** polyarhritis group treated with ferumoxides-labeled BM-MSCs and **c** polyarthritis group treated with ferumoxides-labeled BM-MSCs preconditioned with NaHS. (Scale bar: 50 μm)

To ensure the homing of the infused BM-MSCs and BM-MSCs preconditioned with NaHS into knee joints of the polyarthritis rat model, the cells were labeled prior to injection with ferumoxides. The photomicrograph of the cross-sectioned rat knee joint tissue in the polyarthritis group treated with BM-MSCs not labeled with ferumoxides revealed that there is a negative reaction for Prussian blue staining in the bone trabeculae and bone marrow (Fig. 3a). While, the photomicrograph of the cross-sectioned rat knee joint tissue in the polyarthritis group treated with ferumoxides-labeled BM-MSCs (Fig. 3b) or ferumoxides-labeled BM-MSCs preconditioned with NaHS (Fig. 3c) demonstrated that there are positive reactions for Prussian blue staining in the bone marrow and bone trabeculae.

### Gene expression findings

**Fig. 4:**
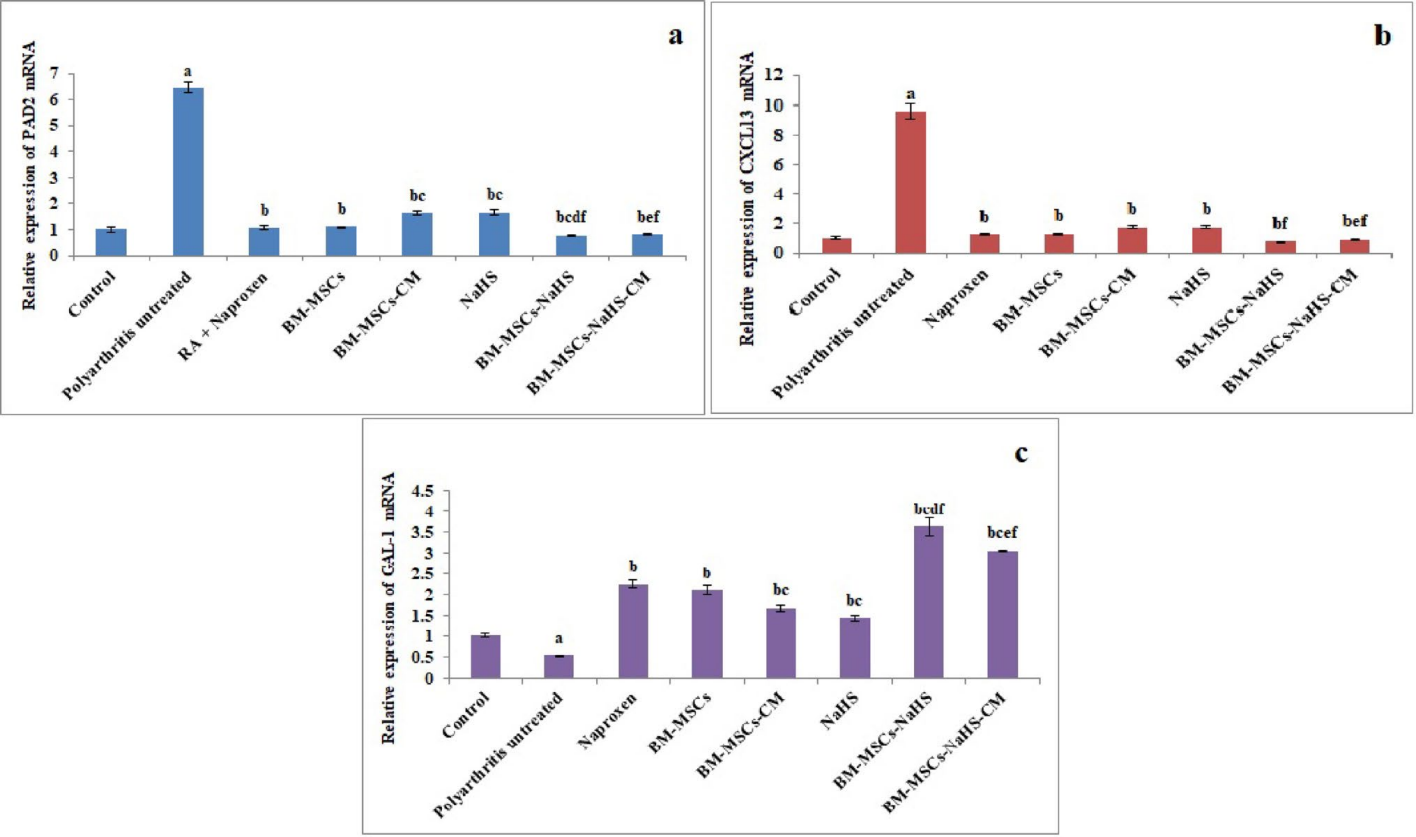
Effect of different treatments on synovial membrane **a** PAD2, **b** CXCL13, and **c** GAL-1 mRNA expression levels in polyarthritis rats. Data are expressed as Means ± S.E of 6 rats/group. ^a^Significant difference at *P <* 0.05 in comparison with the control group. ^b^Significant difference at *P <* 0.05 in comparison with the untreated polyarthritis group. ^c^Significant difference at *P <* 0.05 in comparison with the naproxen group. ^d^Significant difference at *P <* 0.05 in comparison with the BM-MSCs group. ^e^Significant difference at *P <* 0.05 in comparison with the BM-MSCs-CM group. ^f^Significant difference at *P <* 0.05 in comparison with the NaHS group

Figure 4 illustrates the influence of different treatments on synovial membrane PAD2, CXCL13, and GAL-1 mRNA expression levels in polyarthritis rats. Collagen type II injection induced significant (*p* < 0.05) up-regulation of PAD2 and CXCL13 mRNA expression levels in concomitant with significant (*p* < 0.05) down-regulation of GAL-1 mRNA expression level of the synovial membrane by contrast with the control group. On the contrary, the offered treatments for polyarthritis rats triggered significant (*p* < 0.05) down-regulation of PAD2 and CXCL13 mRNA expression levels along with significant (*p* < 0.05) up-regulation of GAL-1 mRNA expression level of synovial membrane *versus* the untreated polyarthritis rats. The groups of rats treated with BM-MSCs-CM or NaHS exhibited significant (*p* < 0.05) up-regulation of PAD2 mRNA expression level associated with significant (*p* < 0.05) down-regulation of GAL-1 mRNA expression level of the synovial membrane relative to that treated with naproxen.

The group of polyarthritis rats treated with BM-MSCs-NaHS disclosed significant (*p* < 0.05) down-regulation of PAD2 mRNA expression level with significant (*p* < 0.05) up-regulation of GAL-1 mRNA expression level of synovial membrane compared to those treated with naproxen, BM-MSCs or NaHS. Also, the rats of the BM-MSCs-NaHS group showed significant (*p* < 0.05) down-regulation of CXCL13 mRNA expression level of the synovial membrane when compared with those of the NaHS group. In like manner, the group of polyarthritis rats treated with BM-MSCs-NaHS-CM exhibited significant (*p* < 0.05) down-regulation of PAD2 and CXCL13 mRNA expression levels with significant (*p* < 0.05) up-regulation of GAL-1 mRNA expression level of the synovial membrane in comparison with those treated with BM-MSCs-CM or NaHS. Furthermore, rats of the BM-MSCs-NaHS-CM group showed significant (*p* < 0.05) up-regulation of GAL-1 mRNA expression level of synovial membrane *versus* those treated with naproxen.

### Protein expression outcomes

**Fig. 5:**
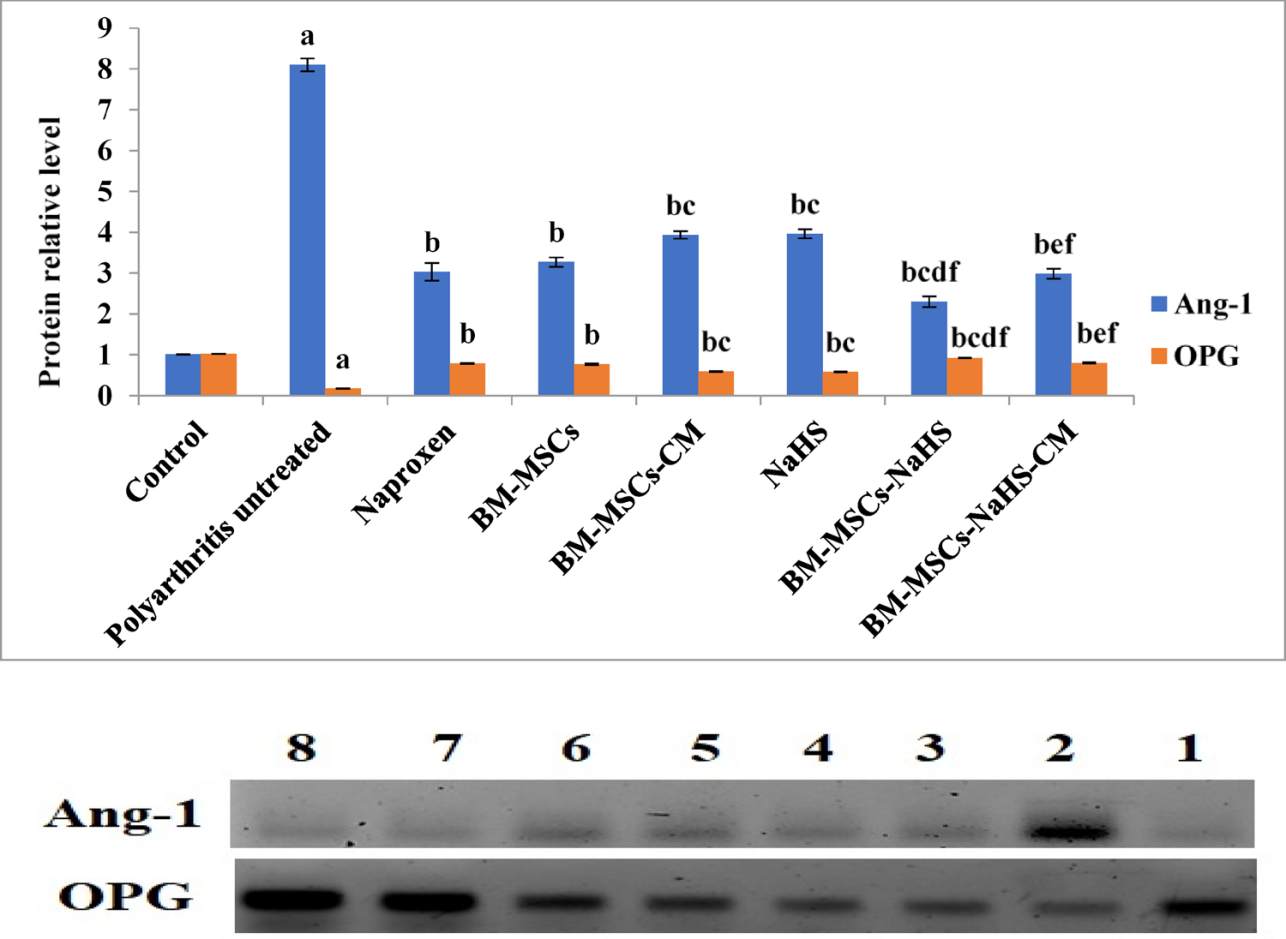
Effect of different treatments on synovial membrane Ang-1 and OPG protein expression levels in polyarthritis rats. (1) Control, (2) polyarthritis untreated, (3) Naproxen, (4) BM-MSCs, (5) BM-MSCs-CM, (6) NaHS, (7) BM-MSCs-NaHS and (8) BM-MSCs-NaHS-CM. Data are expressed as Means ± S.E of 6 rats/group. ^a^Significant difference at *P <* 0.05 in comparison with the control group. ^b^Significant difference at *P <* 0.05 in comparison with the untreated polyarthritis group. ^c^Significant difference change at *P <* 0.05 in comparison with the naproxen group. ^d^Significant difference change at *P <* 0.05 in comparison with the BM-MSCs group. ^e^Significant difference at *P <* 0.05 in comparison with the BM-MSCs-CM group. ^f^Significant difference at *P <* 0.05 in comparison with the NaHS group

Figure 5 clarifies the influence of different treatments on synovial membrane Ang-1 and OPG protein expression levels in polyarthritis rats. Collagen type II injection motivated significant (*p* < 0.05) elevation of synovial membrane Ang-1 protein expression level combined with significant (*p* < 0.05) reduction of OPG protein expression level in contrary to the control group. Whilst, all the applied treatments of polyarthritis rats prompted a significant (*p* < 0.05) reduction of synovial membrane Ang-1 protein expression level along with a significant (*p* < 0.05) elevation of OPG protein expression level by contrast with the untreated polyarthritis rats. Treatment of polyarthritis rats with BM-MSCs-CM or NaHS triggered a significant (*p* < 0.05) increase in synovial membrane Ang-1 protein expression level associated with a significant (*p* < 0.05) drop of OPG protein expression level *versus* those treated with naproxen.

Infusion of BM-MCs-NaHS in polyarthritis rats brought about a significant (*p* < 0.05) decline of synovial membrane Ang-1 protein expression level and a significant (*p* < 0.05) increase of OPG protein expression level when compared with those treated with naproxen, BM-MSCs or NaHS. Moreover, the treatment of polyarthritis rats with BM-MSCs-NaHS-CM induced a significant (*p* < 0.05) decrease in synovial membrane Ang-1 protein expression level and a significant (*p* < 0.05) elevation of OPG protein expression level in comparison with those treated with BM-MSCs-CM or NaHS.

### Histopathological observations

**Fig. 6:**
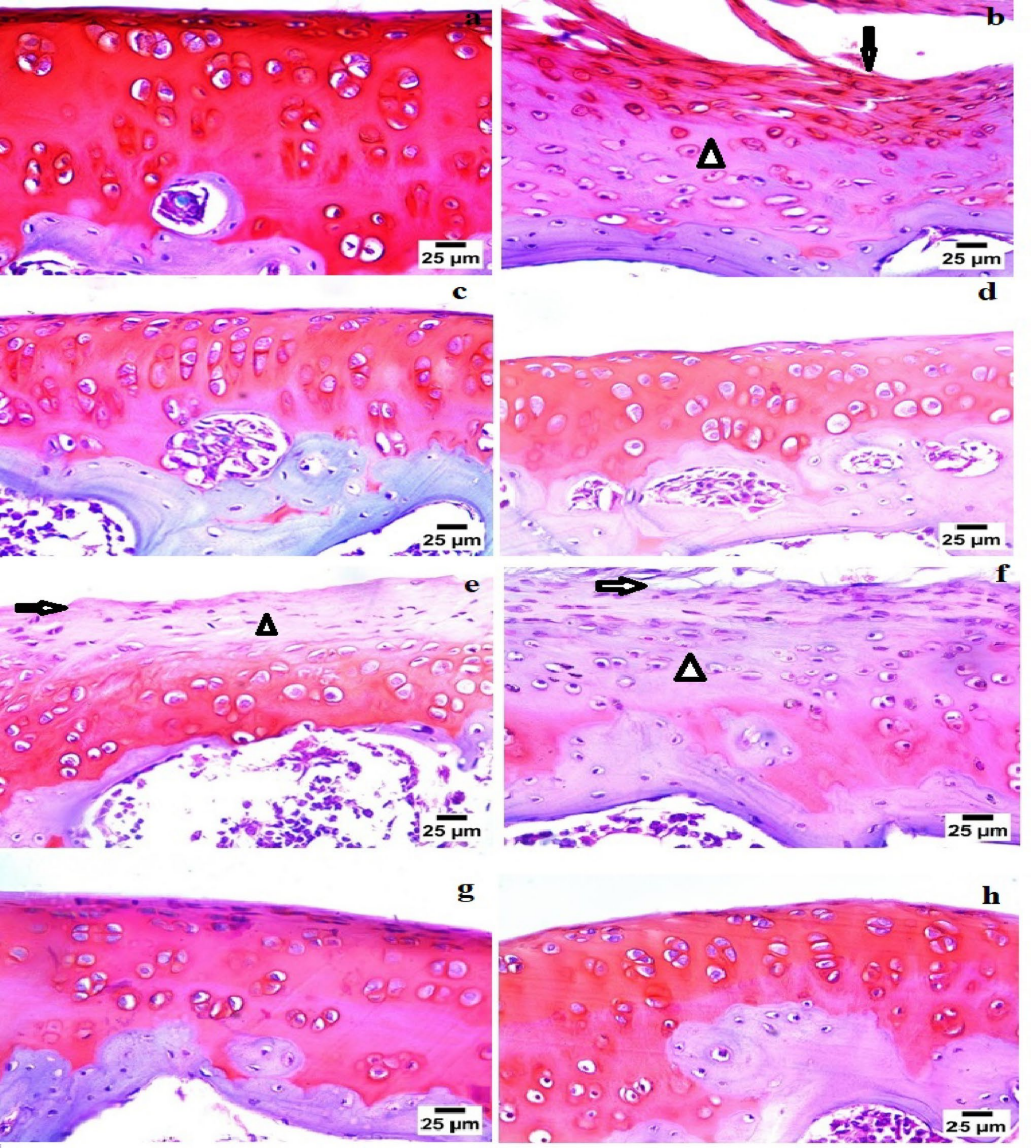
Light microscopy images of safranin O stained knee joints section of rat in **a** control group exhibiting normal histological structure of articular surface with normal proteoglycan, **b** polyarthritis untreated group exhibiting moderate loss of proteoglycan (arrow head) with partial substitution of the articular surface by fibrous connective tissue (arrow), **c** polyarthritis rat treated with naproxen showing the normal histological structure of articular surface with normal proteoglycan, **d** Polyarthritis rat infused with BM-MSCs showing the normal histological structure of articular surface with normal proteoglycan, **e** polyarthritis rat infused with BM-MSCs-CM showing partial loss of proteoglycan (arrow head) with the roughness of articular surface (arrow), **f** polyarthritis rat treated with NaHS showing partial loss of proteoglycan (arrow head) with partial substitution of the articular surface by fibrous tissue (arrow), **g** polyarthritis rat infused with BM-MSCs-NaHS showing normal histological structure of articular surface with normal proteoglycan and **h** polyarthritis rat infused with BM-MSCs-NaHS-CM exhibiting normal histological structure of articular surface with normal proteoglycan

Histopathological examination of the knee joint tissue section of the rat in the control group showed the normal histological structure of the articular surface with normal proteoglycan (Fig. 6a). Nevertheless, histopathological examination of the knee joint tissue section of the rat in the untreated polyarthritis group showed moderate loss of proteoglycan with partial substitution of the articular surface by fibrous connective tissue (Fig. 6b).

The histopathological findings of the knee joint tissue section of polyarthritis rats treated with naproxen or BM-MSCs showed the normal histological structure of the articular surface with normal proteoglycan (Fig. 6c and d respectively). While, histopathological examination of knee joint tissue section of polyarthritis rat infused with BM-MSCs-CM revealed partial loss of proteoglycan with the roughness of articular surface (Fig. 6e). Histological observation of knee joint tissue section of polyarthritis rat administered with NaHS showed there is a partial loss of proteoglycan with partial substitution of the articular surface by fibrous tissue (Fig. 6f). Furthermore, histological examination of knee joint tissue section of polyarthritis rats infused with BM-MSCs-NaHS or BM-MSCs-NaHS-CM indicated normal histological structure of articular surface with normal proteoglycan (Fig. 6g and h respectively).

## Discussion

In this approach, BM-MSCs were isolated from male rats, cultured, and characterized by their adhesion properties and fusiform shape, as well as the excitence of typical surface markers CD90^+^ and CD105^+^ and the lack of the hematopoietic marker CD45^−^ (Mahmoud et al. 2020; Aglan et al. 2024). Moreover, BM-MSCs and those preconditioned with NaHS were detected in damaged knee joints, as evidenced by the positive Prussian blue staining. This supports the idea that allogeneic or xenogeneic BM-MSCs can integrate into the host after infusion. This finding suggests that BM-MSCs can migrate to inflammatory sites and infiltrate knee joint tissue following intravenous injection, thereby inhibiting joint inflammation and modulating immune responses (El Qashty et al. 2018; Liu et al. 2018). Moreover, MSCs are known to express a wide range of chemokines and receptors, forming a complex chemotactic network in vivo to direct circulating cells to the injured sites or mobilize immune cells within the inflammatory tissues (Andreas et al. 2014).

In this study, two therapeutic agents were utilized; NaHS and BM-MSCs. Each exerts its therapeutic effect through different mechanisms: NaHS serves as a hydrogen sulfide (H₂S) donor, and H₂S is the active component responsible for anti-inflammatory, antioxidant, and pro-regenerative effects via signaling pathways. On the other hand, BM-MSCs are living cellular therapeutics. Their efficacy is not attributed to a single active molecule but to a combined action involving paracrine signaling, secretion of anti-inflammatory cytokines (e.g., IL-10, TGF-β), immunomodulation, and the release of extracellular vesicles that promote tissue repair and modulate immune responses. Given the complexity of MSCs and the established effective dose of NaHS based on previous studies (Fang et al. 2009; Abdelmonem et al. 2019; Haikal et al. 2019), the current experiment used a single therapeutic dose per group to compare efficacy among treatment strategies.

The collagen-induced arthritis (CIA) model is a well-established experimental model that mimics many clinical, histopathological, and immunological features of human rheumatoid arthritis (RA) (Wooley 2004). Because of these similarities, the CIA model is useful for predicting how effective therapy might be in humans (Hegen et al. 2008; Baddack et al. 2013). The current investigation demonstrated that injecting collagen type II in rats leads to a significant increase in the gene expression level of PAD2 in the synovial membrane which is in consistent with the finding of Vossenaar et al. (2003). PAD enzymes catalyze the calcium-dependent deamination of arginine residues and produce citrulline (non-classical amino acid) (Curran et al. 2020). Peptidyl arginine deiminases (PADs) are involved in the pathogenesis of seropositive RA because they generate citrullinated proteins, which are recognized by the anti-citrullinated protein antibodies (ACPAs). PAD2 and PAD4, among the five PAD enzymes, are the most significantly implicated in the pathogenic citrullination process in RA (Rebernick et al. 2017). Soon after ACPAs are identified, PAD2 and PAD4 are expressed by infiltrating hematopoietic cells like monocytes, granulocytes, and macrophages in the RA synovial membrane (Chang et al. 2009). PAD2 is widely distributed in the body, including in leukocytes and it is mainly located in the cytosol and lacking a conventional nuclear localization signal, but it can move to the nucleus and be spontaneously released by neutrophils (Zhang et al. 2012; Zhou et al. 2017). Being calcium-dependent, PAD2 becomes overactive in conditions with elevated Ca^+ 2^ levels, such as during apoptosis or terminal epidermal differentiation, particularly in the vicinity of PAD-containing macrophages (antigen-presenting cells, APCs) (Slade et al. 2015). Upon activation in macrophages, particularly in response to lipopolysaccharides (LPS) (Yu and Proost 2022), toll-like receptors (TLRs), such as TLR4, are upregulated, leading to the overexpression and activation of PAD2 (Malmström et al. 2017; Romão and Fonseca 2022). In a study using a mouse model of arthritis, PAD2 appears to be crucial for sustaining significant levels of citrullination in inflammatory arthritis (Rebernick et al. 2017). This hypothesis evidenced the observation that the activity of PAD enzymes in synovial fluid is associated with the levels of PAD2 (Damgaard et al. 2014). These changes are linked with ACPA positivity, disease activity, and concentrations of inflammatory mediators (Damgaard et al. 2016).

The present data from the groups treated with naproxen, BM-MSCs, BM-MSCs-CM, NaHS, or BM-MSCs pre-conditioned with NaHS as well as their conditioned media all demonstrated a significant down-regulation of the PAD2 gene expression level. The group treated with naproxen showed a significant decrease in the PAD2 gene expression level, which is likely due to naproxen’s ability to indirectly decrease the LPS-induced formation of PGE_2_ (Bertin et al. 1994). The relationship between PGE_2_ and macrophage polarization was demonstrated by Sheppe et al. (2018) who observed that exposing macrophages to high doses of PGE_2_ seemed to tip the balance towards the M1 (pro-inflammatory) phenotype in response to *Salmonella enterica* Typhimurium infection. The reduction in PAD2 expression levels subsequently impacts macrophage polarization, leading to a shift in the M1/M2 phenotype towards the M2 (anti-inflammatory) phenotype.

The significant down-regulation of PAD2 mRNA expression level in the rat group treated with BM-MSCs is mainly induced through monocyte/macrophage modulation. MSCs inhibit monocyte differentiation (Spaggiari et al. 2009), in addition to switching macrophages from the M1 to the M2 (Jiang and Xu 2020). MSCs could also repress interleukin-1 receptor antagonist (IL-1RA) to suppress the response of the immune system and raise M2 macrophage proliferation (Luz-Crawford et al. 2016). Furthermore, BM-MSCs could produce interleukin-10 (IL-10), which hampers APCs maturation (Moore et al. 2001), and directs macrophage proliferation toward the M2 phenotype (Smallie et al. 2010).

The function of NaHS as an H_2_S donor in reducing the PAD2 mRNA expression level significantly in the rat group treated with NaHS has recently been elucidated through its capacity to transition macrophage phenotype from pro-inflammatory M1 to anti-inflammatory M2 (Du et al. 2014) prompting the synthesis of the anti-inflammatory cytokine IL-10 (Li et al. 2009). Additionally, H_2_S diminishes the expression of TLR4, which is heightened by LPS stimulation (Zhang et al. 2018), resulting in a down-regulation of the PAD2 gene expression level.

CXCL13, also called B-lymphocyte chemoattractant (BLC) or C-X-C motif chemokine 13, plays a pivotal role in guiding and activating cells within lymphoid and non-lymphoid tissues. Heightened CXCL13 expression in non-lymphoid tissues promotes B-cell migration and the formation of ectopic lymphoid tissues (Yoshitomi 2020). Its production by a variety of cells in the synovium, such as T cells, monocytes/macrophages, endothelial cells, and fibroblasts, is well-documented (Drosos et al. 2020). This chemokine interacts with its receptor, CXCR5, to attract B cells and follicular T-helper cells expressing CXCR5 (Humby et al. 2009). Its interaction between CXCL13 and CXCR5 is crucial for B cell differentiation into plasma cells and subsequent antibody production (Wengner et al. 2007). Tsai et al. (2021) investigated the role of the CXCL13/CXCR5 axis in endothelial progenitor cell homing and angiogenesis during RA using a CIA mouse model. The results of that study revealed that heightened CXCL13 levels in RA patients’ synovial fluid attract endothelial progenitor cells, promoting angiogenesis in inflamed joints—a pivotal event in disease progression—through CXCR5 receptor interactions. The current finding comes in line with Tsai et al. 2021 research, demonstrating a significant increase in CXCL13 mRNA expression level in the synovial membrane of rats injected with collagen type II compared to controls.

Treating polyarthritis rats with naproxen, BM-MSCs, BM-MSCs-CM, NaHS, BM-MSCs-NaHS, or BM-MSCs-NaHS-CM resulted in a significant decrease in CXCL13 mRNA expression level in the synovial membrane. The reduced levels of CXCL13 gene expression in the naproxen-treated group could be explained by the inhibition of nuclear factor kappa B (NF-κB) activity within IL-1β-induced chondrocytes (Cheleschi et al. 2015) and the decrease of IL-6 production by synovial tissue fibroblasts (Pelletier et al. 1997). It is well known that tumor necrosis factor alpha (TNF-α) and IL-6 can induce CXCL13 expression, fostering the evolution of B cell follicles and germinal center reactions in the synovium (Meeuwisse et al. 2011).

The decrease in CXCL13 gene expression levels observed after BM-MSC infusion in polyarthritis rats shows parallelism with the findings of Gowhari Shabgah et al. (2019). MSCs can reduce the migratory capacity of pathogenic B cells by secreting IL-10, transforming growth factor beta (TGF-β), nitric oxide (NO), and indoleamine 2,3-dioxygenase (IDO). These factors also influence cell proliferation and differentiation, immunoglobulin production, and the expression of CXCR5 (Lucero and Castro-manrreza 2021). The indirect impact of H_2_S donors on CXCL13 gene expression could be attributed to their ability to regulate the expression of various pro-inflammatory cytokines, chemokines, and enzymes, largely through the modulation of NF-κB activity (Sunzini et al. 2020). Furthermore, H_2_S has a vital function in coordinating the recruitment and infiltration of immune cells into tissues, inhibiting leukocyte migration via direct suppression of the adherence of circulating cells to inflamed vascular walls. Consequently, H_2_S can reduce the infiltration of neutrophils and lymphocytes into tissues (Zanardo et al. 2006).

The obtained data from the groups treated with BM-MSCs-CM or BM-MSCs-NaHS-CM indicated a decrease in CXCL13 gene expression level that can be explained by numerous experimental researches on MSC-EVs as cell-free medication for arthritis in animal models which were summarized by Miao 2022. It has been cited that, cell culture-conditioned medium is a valuable source of EVs for basic scientific, therapeutic, and diagnostic applications (Shekari et al. 2023). BM-MSC-EVs can diminish the progress of osteoarthritis by mitigating the damage of the cartilage, minimizing osteophyte development and synovial macrophage infiltration (Lukomska et al. 2019; Zhang et al. 2020), which are considered to be the main sources of CXCL13. In addition, the synovial fluid expression levels of IL-1, IL-6, and TNF-α were depressed by BM-MSCs-EVs. Moreover, MSC-EVs containing miRNA-150-5p inhibited angiogenesis and fibroblast-like synovitis (FLS) cell proliferation in patients with RA and model of CIA by down-regulating vascular endothelial growth factor (VEGF) (Chen et al. 2018) which antagonizes CXCL13 effect in synovial tissue (Tsai et al. 2021).

The examination of GAL-1 gene expression level in the synovial membrane of the polyarthritis group displayed significant down-regulation vresus the control group, aligning with the findings of Ohshima et al. (2003). Galectin-1, identified as the first galectin, typically acts as a mediator for pro-resolution by inhibiting various innate and adaptive immune processes. Structurally, GAL-1 consists of two subunits of 14.5 kDa (135 amino acids), existing in a dynamic equilibrium of dimerization (Méndez-Huergo et al. 2017). Due to an atypical count of six cysteine residues, this lectin is particularly prone to oxidative inactivation, which restricts its biological function (Di Lella et al. 2011). Although traditionally seen as separate processes, research suggests that these mode of actions may be interlinked, as dimerization enhances ligand binding, thereby shielding GAL-1 from oxidative inactivation (Stowell et al. 2009). The sensitivity to GAL-1 is affected by intrinsic and extrinsic factors, including dimerization equilibrium, redox status, and the controlled activity of glycosyltransferases that either create or hinder specific glycan structures on target cells (Cerliani et al. 2017). Within the immune system, GAL-1 is produced and released by various cells, including activated T and B cells (Fuertes et al. 2004), macrophages (Rabinovich et al. 1996), fork-head box p3 (Foxp3) regulatory T cells (Tregs), tolerogenic dendritic cells (DCs) (Ilarregui et al. 2009; Tesone et al. 2016), γ, δ T cells (Rutkowski et al. 2015), microglia (Starossom et al. 2012), and myeloid-derived suppressor cells(Rutkowski et al. 2015).Its expression is significantly altered in inflammatory conditions (Davicino et al. 2017), autoimmunity (Ilarregui et al. 2009), and allergy (Xie et al. 2017). Notably, in the experimental models, the expression of GAL-1 peaks during the recovery phase of autoimmune diseases (Starossom et al. 2012), suggesting a significant role for this lectin in inflammation resolution.

Galectin-1 modulates immune responses via surface receptor binding. It regulates the negative selection of T cells in the thymus (Liu et al. 2008), promotes apoptosis in Th1 and Th17 cells (Toscano et al. 2007), and induces the shift from Th1 to Th2 polarized immune responses (Rabinovich et al. 1999a). Treatment of T cells with GAL-1 alters the cytokine profile, leading to depressed levels of pro-inflammatory cytokines such as TNFα, IL-1β, IL-2, and interferon-gamma (IFNγ) (van der Leij et al. 2004) and elevated the levels of anti-inflammatory cytokines such as IL-10 (van der Leij et al. 2007). In B cells, GAL-1 negatively regulates cell proliferation and B cell receptor (BCR)-mediated signal transduction (Yu et al. 2006). Galectin-1 also modulates the activation of innate immune cells; treatment with GAL-1 markedly reduces neutrophil infiltration, mast cell degranulation (Rabinovich et al. 2000), and expression of inducible nitric oxide synthase (iNOS) in macrophages (Correa et al. 2003). The association between GAL-1 and RA was first documented by Rabinovich et al. (1999b) using a CIA mouse model. A single injection of fibroblasts engineered to secrete mouse GAL-1 or daily supplementation of 100 µg of recombinant human GAL-1 in DBA/1 mice was suitable to inhibit the overall clinical and histopathological manifestations of CIA. Galectin-1 therapy also depleted anti-collagen antibody levels and shifted the cytokine profile towards a type-2 polarized immune reaction. Further exploration of the mechanism indicated that GAL-1 medication enhances the susceptibility of T cells to antigen-induced apoptosis, augments T cell adhesion to the extracellular matrix, and inhibits IL-2 secretion from collagen-specific T cell hybridomas (Rabinovich et al. 1999; van der Leij et al. 2004; Toscano et al. 2007). It has been demonstrated that, leukocyte adhesion and emigration were notably elevated in GAL-1-deficient mice inflamed with IL-1β (Cooper et al. 2008). Moreover, it has been reported that, the decreased levels of synovial fluid GAL-1 correlate with the heightened levels of anti-GAL-1 autoantibodies and anti-cyclic citrullinated peptide (CCP) antibodies in RA (Xibillé-Friedmann et al. 2013).

Treatment of polyarthritis rats with naproxen, BM-MSCs, BM-MSCs-CM, NaHS, BM-MSCs-NaHS, or BM-MSCs-NaHS-CM produced a significant elevation in the mRNA expression level of the synovial membrane GAL-1 gene. The increase in GAL-1 gene expression upon naproxen treatment can be explained by the findings of Abramson and Weissmann (1989). Naproxen, like other NSAIDs, can block one or both cyclooxygenase (COX) isoforms. COX, particularly COX-2, is directly linked to the production of reactive oxygen species (ROS), and vice versa, indicating a connection between oxidative stress and inflammation-mediated induction of COX. The reduction in oxidative stress enhances GAL-1 activity, leading to an up-regulation of GAL-1 gene expression level (Aletaha et al. 2010b).

The observed up-regulation in the GAL-1 gene expression level after infusion of BM-MSCs and BM-MSCs-CM in polyarthritis rats is consistent with the results published by Sioud et al. (2011). These investigators demonstrated that BM-MSCs express and secrete GAL-1 in the culture supernatants, where it functions as an extracellular protein to activate cells and mediate cell-cell and cell-ECM interactions. On the other hand, the effect of NaHS on the up-regulation of GAL-1 expression level arises from the role of H_2_S in balancing oxidative and reductive species, thereby affecting the cell’s redox state. H_2_S is a strong reducing agent that can directly react with multiple oxidative stressors, including superoxide radical anion (Mitsuhashi et al. 2005), hydrogen peroxide (Geng et al. 2004), and peroxynitrite (ONOO^−^) (Whiteman et al. 2004). Additionally, H_2_S can enhance the activity of key enzymes involved in the cell’s antioxidant defense. It has also been found that H_2_S can stimulate FOXP3 activation leading to the differentiation of T regulatory cells (Yang et al. 2015), which are known to highly express GAL-1 (Dalotto-Moreno et al. 2013).

The current study demonstrated that injecting collagen type II into rats significantly increased the synovial membrane’s Ang-1 protein expression levels. This finding is in conformity with that published by Ao et al. (2022) who examined the effect of Martine (Mat) on reducing Ang-1, Ang-2, and Tie-2 protein expression in polyarthritis rats. Angiogenesis is a complex process involving the proliferation and migration of endothelial cells and the formation of primitive vascular tubes. Early stages are mediated by angiogenic factors such as VEGF and basic fibroblast growth factor (bFGF), while Ang-1 is essential for the stabilization and maturation of blood vessels, requiring the recruitment and differentiation of MSCs into vascular smooth muscle cells. Unlike other angiogenic factors, Ang-1 does not significantly stimulate endothelial growth but is a strong chemoattractant that enhances endothelial migration (Witzenbichler et al. 1998). Angiopoietin-1 also promotes endothelial cell adhesion to fibronectin and prevents endothelial cell apoptosis (Huang et al. 1999). Overexpression of Ang-1 increases the number of blood vessels, demonstrating its proangiogenic effect (Suri et al. 1998). Previous studies have shown that Ang-1 expression is regulated differently in RA synovial tissue fibroblasts compared to endothelial cells; for example, TNF-α significantly increases Ang-1 levels in RA fibroblasts while not affecting endothelial Ang-1 levels (Gravallese et al. 2003).

Treatment polyarthritis rats with naproxen, BM-MSCs, BM-MSCs-CM, NaHS, or BM-MSCs pre-conditioned with NaHS, as well as their conditioned media elicited significant down-regulation of synovial membrane Ang-1 protein expression with variable degrees. The suppression of TNF-α level as shown in our results obtained after applying the proposed treatments (data not shown), may be the cause of down-regulating Ang-1 protein expression as Ang-1 protein is mainly induced in synovial fibroblasts by TNF-α via the NF-κB pathway (Gravallese et al. 2003; Scott et al. 2005).

The data of the current work demonstrated that, injection of collagen type II in rats motivated a significant diminution of synovial membrane OPG protein expression matching the result recorded by Li et al. (2021). Osteoprotegerin, is a member of the TNF receptor superfamily, derived from osteoblasts as a soluble decoy receptor for the osteoclast differentiation factor receptor-activator of nuclear factor κB ligand (RANKL) (Komatsu and Takayanagi 2018). Osteoprotegerin principally suppresses the RANKL/RANK signaling pathway activity by competing with RANK on the surface of osteoclast precursor cells to bind RANKL (Metzger and Narayanan 2019) to inhibit bone resorption (Komatsu and Takayanagi 2018). Furthermore, the RANKL/OPG/RANK axis has been found to regulate bone remodeling (Whyte et al. 2002). Fadda et al. (2015) stated that the alternations in the RANKL/OPG system, involving an increase in RANKL and a decrease in OPG in the peripheral blood and synovial tissue, constitute a fundamental mediator of bone resorption in RA-induced osteoporosis.

Treatment of polyarthritis rats with naproxen, BM-MSCs, BM-MSCs-CM, NaHS, BM-MSCs-NaHS, or BM-MSCs-NaHS-CM significantly up-regulated protein expression of synovial membrane OPG. The increased OPG expression due to naproxen treatment could be attributed to naproxen’s ability to reduce RANKL levels. Generally, RANKL down-regulation is associated with OPG up-regulation (Hofbauer and Schoppet 2004). This is achieved through the reduction of elevated COX-2 expression and COX-induced PGE_2_ production (Brutzkus et al. 2023). To understand this indirect mechanism, it is essential to define the relationship between COX-2, PGE_2_, osteoclastogenesis, and bone resorption. In response to inflammatory and stress signals, osteoblasts induce COX-2 expression via extracellular signal-regulated kinase1/2 mitogen-activated protein (ERK1/2 MAP) kinase, p38 MAP kinase, and NF-κB-mediated mechanisms. This induces the production of PGE_2_, which binds to the PGE_2_ receptor 4 (EP4) receptor in osteoblasts through autocrine/paracrine mechanisms, leading to the significant expression of RANKL in osteoblasts. PGE_2_-mediated RANKL expression ultimately induces osteoclastogenesis from monocyte-macrophages in bone leading to the remarkable advancement of osteoclastogenesis and bone resorption (Ohshiba et al. 2003).

The observed up-regulation in the OPG protein expression after infusion of BM-MSCs or BM-MSCs-CM in polyarthritis rats is in agreement with Oshita et al. (2011) who demonstrated the role of MSCs in inhibiting osteoclastogenesis and suppressing osteoclast differentiation upraised from constitutive generation of OPG under cell–cell contact–free conditions. On the other hand, the effect of NaHS in up-regulating OPG protein expression corresponds to the results of Behera et al. (2018) who proved the effect of NaHS in mitigating bone loss via the regulation of OPG/RNKL through C-Jun/JNK-p signaling pathway. The activated C-Jun/JNK-p signaling through oxidative stress results in activated JNK-p that regulates DNA methyl transferase 1 (DNMT1) expression through binding to its promoter and reinorces OPG hyper-methylation, leading to osteoblast dysfunction. Administration of NaHS reverses these changes and upregulates OPG level.

The aforementioned data were confirmed by the histopathological examination of rats’ knee joint sections in the different studied groups using safranin O that stains proteoglycans in the cartilage matrix. The microscopic image revealed significantly low color density in the polyarthritis group compared to the control group referring to loss of proteoglycan in the cartilage matrix which agrees with Young et al. (2006) and Elsaid et al. (2009). The degration of the cartilage matrix components are accounted both as diagnostic markers of cartilage damage and as potential autoantigens in the induction and maintenance of RA synovial inflammation (Otero and Goldring 2007). Cartilage proteoglycan is a mucinous glycoprotein liberated from fibroblast-derived type, B synoviocytes and chondrocytes (Jay et al. 1998). It engages to joint lubrication, inhibits friction between cartilage surfaces, and prevents synovial overgrowth (Damen et al. 2021). It is able to bind in a specific fashion to hyaluronic acid and such association produces large multimolecular aggregates, which are maintained within the extracellular matrix by a network of type II collagen (Wall and Board 2014). Cartilage matrix molecules provide cushioning of the cartilage in the joint. Loss of ground substance in turn allows a greater susceptibility to damage from compressive forces and greater penetration of degenerative molecules leading to surface corrosions (Moody et al. 2018). Synthesis of proteoglycan and its gene expression by chondrocytes is induced by TGF-β and depressed after exposure to IL-1β and TNF-α (Schmidt et al. 2008; Cuellar and Reddi 2014).

Microscopic images of knee joint tissue section from polyarthritis rats treated with naproxen showed a normal histological structure of the articular surface with intact proteoglycan, likely attributed to naproxen’s ability to reduce the generation of inflammatory cytokines such as TNF-α and IL-β (Dief et al. 2015; Hsueh et al. 2020). These cytokines are known to inhibit cartilage proteoglycan (Schmidt et al. 2008; Cuellar and Reddi 2014).

Similarly, the microscopic image of the knee joint tissue section from polyarthritis rats treated with BM-MSCs revealed a normal histological structure of the articular surface with preserved proteoglycan which is consistent with the findings of Mohammed et al. (2018). This outcome is likely attributed to the reduction in inflammatory cytokines like TNF-α and the production of anti-inflammatory modules such as TGF-β, which influence matrix turnover and inhibit proteoglycan degradation (Kehoe et al. 2014). Meanwhile, the microscopic image of the knee joint tissue section of polyarthritis rats after treatment with BM-MSCs-CM revealed partial loss of proteoglycan with roughness of the articular surface. The main cause of the superior effect of BM-MSCs rather than their conditioned media in the inflamed tissue recovery is that, despite the ability of BM-MSCs to secrete different anti-inflammatory molecules that enhance the cartilage matrix refine specially TGF-β (Mattar and Bieback 2015; Najar et al. 2016), the direct cell contact has a potential role in the regenerative capacity of BM-MSCs for cartilage matrix repair (Jiang and Xu 2020).

In contrast, the microscopic image of the knee joint tissue section from polyarthritis rats treated with NaHS showed partial substitution of the articular surface by fibrous tissue. It has been recorded that, exogenous H_2_S therapy inhibited inflammation and oxidative stress, ameliotrated skeletal muscle fibrosis, and partly improved skeletal muscle injury (Zhao et al. 2020). Interestingly, the microscopic images of knee joint tissue sections of polyarthritis rats infused with BM-MSCs-NaHS or their CM indicated the normal histological structure of articular surface with normal proteoglycan.

The above-mentioned outcomes proved the potential therapeutic efficacy of BM-MSCs preconditioned with NaHS over BM-MSCs or NaHS alone and the potent therapeutic impact of BM-MSCs preconditioned with NaHS-CM over the BM-MSCs-CM alone in the treatment of polyarthritis-modeled rats. Preconditioning BM-MSCs with NaHS enhances their therapeutic potential by promoting cell survival, proliferation, and resistance to oxidative stress (Zhang et al. 2016; Mohammed et al. 2021). NaHS-preconditioned BM-MSCs exhibit increased secretion of bioactive molecules such as IL-10, TGF-β, and PGE2, which collectively suppress the activation of proinflammatory T cells and macrophages while enhancing regulatory T cell formation (Shimizu et al. 2022; Ntege et al. 2020). This paracrine effect contributes to the restoration of immune homeostasis within the inflamed synovial microenvironment. Additionally, NaHS preconditioning improves the homing ability of BM-MSCs to damaged tissues, further augmenting their regenerative and anti-angiogenic effects (Zhang et al. 2019; Goren et al. 2019a, b).These multifaceted actions of H₂S potentiate the efficacy of BM-MSC-based therapies in mitigating chronic inflammation and joint destruction characteristic of polyarthritis (Mohammed et al. 2021; Song et al. 2024).

In conclusion, NaHS modulates the therapeutic potential of BM-MSCs in a polyarthritis rat model by reducing angiogenesis, ectopic lymphoid tissue formation, and osteoclastogenesis as evidenced by the decrease in PAD2 and CXCL13 mRNA expression levels as well as Ang-1 protein expression in synovial membrane along with the increase in GAL-1 mRNA expression level and OPG protein expression. This approach of using BM-MSCs preconditioned with NaHS may have a prominent impact on the treatment of RA patients in the future.

While this study compared the efficacy of single standardized doses of NaHS and BM-MSCs-based therapies, future investigations should incorporate multiple dose levels of each agent to establish dose–response relationships, as recommended by regulatory standards.

## Data Availability

The datasets used and/or analyzed during the current study are available from the corresponding author on reasonable request.
